# Phylogeography of the Koala, (*Phascolarctos cinereus*), and Harmonising Data to Inform Conservation

**DOI:** 10.1371/journal.pone.0162207

**Published:** 2016-09-02

**Authors:** Linda E. Neaves, Greta J. Frankham, Siobhan Dennison, Sean FitzGibbon, Cheyne Flannagan, Amber Gillett, Emily Hynes, Kathrine Handasyde, Kristofer M. Helgen, Kyriakos Tsangaras, Alex D. Greenwood, Mark D. B. Eldridge, Rebecca N. Johnson

**Affiliations:** 1 Australian Centre for Wildlife Genomics, Australian Museum Research Institute, 1 William Street, Sydney, New South Wales, 2010, Australia; 2 Royal Botanic Garden Edinburgh, 20A Inverleith Row, Edinburgh, EH3 5LR, United Kingdom; 3 School of Agriculture and Food Science, The University of Queensland, Brisbane, Queensland, 4072, Australia; 4 Koala Hospital Port Macquarie, PO Box 236, Port Macquarie, NSW, 2444, Australia; 5 Australia Zoo Wildlife Hospital, Beerwah, Queensland, 4519, Australia; 6 Ecoplan Australia Pty Ltd, PO Box 968 Torquay, Victoria, 3228, Australia; 7 School of BioSciences, The University of Melbourne, Victoria, 3010, Australia; 8 National Museum of Natural History, Smithsonian Institution, Washington, DC, United States of America; 9 Department of Translational Genetics, The Cyprus Institute of Neurology and Genetics, 6 International Airport Ave., 2370 Nicosia, Cyprus; 10 Leibniz Institute for Zoo and Wildlife Research, 10315, Berlin, Germany; 11 Department of Veterinary Medicine, Freie Universität Berlin, 14163, Berlin, Germany; Australian National University, AUSTRALIA

## Abstract

The Australian continent exhibits complex biogeographic patterns but studies of the impacts of Pleistocene climatic oscillation on the mesic environments of the Southern Hemisphere are limited. The koala (*Phascolarctos cinereus*), one of Australia’s most iconic species, was historically widely distributed throughout much of eastern Australia but currently represents a complex conservation challenge. To better understand the challenges to koala genetic health, we assessed the phylogeographic history of the koala. Variation in the maternally inherited mitochondrial DNA (mtDNA) *Control Region* (*CR*) was examined in 662 koalas sampled throughout their distribution. In addition, koala *CR* haplotypes accessioned to Genbank were evaluated and consolidated. A total of 53 unique *CR* haplotypes have been isolated from koalas to date (including 15 haplotypes novel to this study). The relationships among koala *CR* haplotypes were indicative of a single Evolutionary Significant Unit and do not support the recognition of subspecies, but were separated into four weakly differentiated lineages which correspond to three geographic clusters: a central lineage, a southern lineage and two northern lineages co-occurring north of Brisbane. The three geographic clusters were separated by known Pleistocene biogeographic barriers: the Brisbane River Valley and Clarence River Valley, although there was evidence of mixing amongst clusters. While there is evidence for historical connectivity, current koala populations exhibit greater structure, suggesting habitat fragmentation may have restricted female-mediated gene flow. Since mtDNA data informs conservation planning, we provide a summary of existing *CR* haplotypes, standardise nomenclature and make recommendations for future studies to harmonise existing datasets. This holistic approach is critical to ensuring management is effective and small scale local population studies can be integrated into a wider species context.

## Introduction

The Australian continent exhibits complex biogeographic patterns as a consequence of the interplay between continental drift, topography and climatic change [1–3]. In contrast to the Northern Hemisphere, where continental ice sheets restricted species to relatively few major refugia during glacial cycles [4], the Southern Hemisphere is characterised by biogeographic barriers which periodically hampered or prevented gene flow/movement during periods of increased aridity. While multiple biogeographic barriers have been identified across Australia, these often appear idiosyncratic, with impacts on the phylogeographic patterns varying according to species-specific characters such as dispersal ability and habitat requirements [1,5–7]. As a result, biogeographic barriers are frequently detected in some species but not others, and the limited number of studies restricts our understanding of the impacts of past climate change on the Australian flora and fauna.

**Fig 1:**
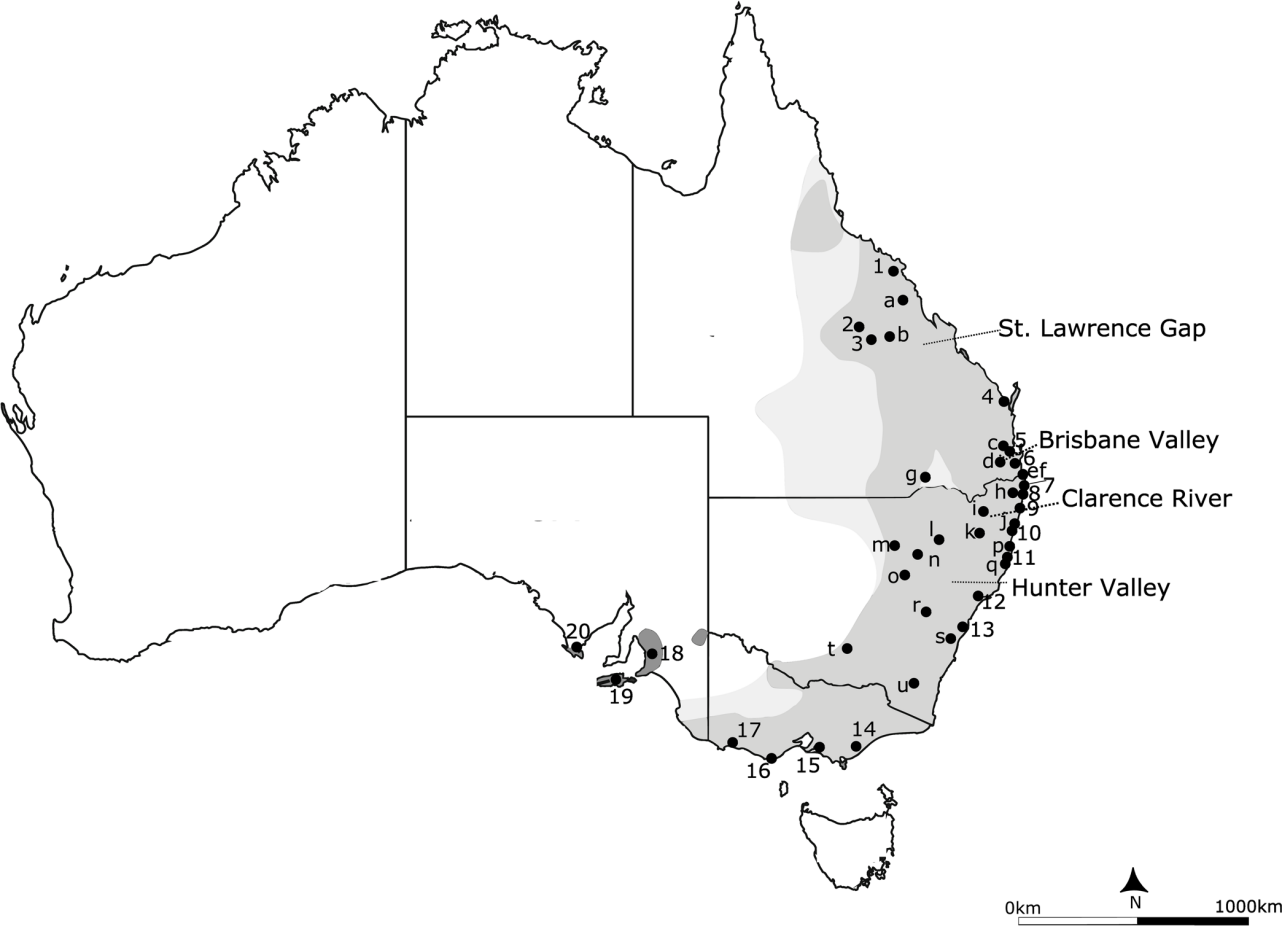
Geographical distribution of the koala, *Phascolarctos cinereus*, showing the sampling locations. The current distribution is shown in grey, with the historical range in light grey and introduced populations in dark grey. Sampling locations are 1, Whitsunday/Mackay; 2, Blair Athol; 3, Clermont; 4, Maryborough; 5, Redlands; 6, Coomera; 7, Tyagarah; 8, Ballina; 9, Iluka; 10, Pine Creek; 11, Port Macquarie; 12, Maitland; 13, Campbelltown; 14, Strzelecki/East Gippsland; 15, French Island; 16, Cape Otway; 17, Bessiebelle; 18, Mt. Lofty Ranges; 19, Eyre Peninsula; 20, Kangaroo Island; a, Isaac Region; b, Peak Downs; c, Brookfield; d, Peak Crossing; e, Tweed Heads; f, Tanglewood; g, Balonne/Goondiwindi; h, Lismore; i, Glen Innes; j, Grafton; k, Armidale; l, Gunnedah; m, Coonamble; n, Coonabarabran; o Dubbo; p, Kempsey; q, Dunbogan; r, Bathurst; s, Mittagong; t, Narrandera; u, Bredbo. Sampling locations 1–20 contain at least 7 individuals, while sites a-u contain fewer individuals and/or individuals were widely dispersed over a large area. Putative biogeographic barriers Brisbane Valley and Clarence River, St. Lawrence Gap and Hunter Valley are shown as dashed lines.

The koala (*Phascolarctos cinereus*) is currently widely distributed throughout eastern Australia, from north Queensland to South Australia, including several insular populations (Fig 1; [8]). The mainland range encompasses several putative biogeographic barriers, including the St Lawrence Gap (SLG), the Brisbane Valley Barrier (BVB), the Clarence River Barrier (CRB) and the Hunter Valley Barrier (HVB). Koalas also have a complex management history, with numerous translocations (sometimes not comprehensively documented) having occurred throughout the southern parts of their range since the 1920s [8]. In Victoria (Vic), small numbers of koalas were introduced onto offshore islands, notably French and Phillip Islands. These populations were subsequently used as a source for reintroductions on mainland Vic, and potentially also in western New South Wales (NSW; [9]). In South Australia (SA), koalas were extinct by the 1930s [10,11] but populations in the Mt Lofty Ranges were established using koalas from Queensland (Qld), NSW and Vic, and this population was subsequently used to establish other populations on mainland SA [10,12,13]. Additionally, koalas were introduced to Kangaroo Island, SA from French Island, and from there subsequently used to establish populations on Eyre Peninsula, SA [10,12,13].

Koalas exhibit substantial morphological variation throughout their range, which appears to reflect clinal variation in response to environmental conditions (e.g. size and colouration; [12,14–16]). Initially, three koala subspecies were recognised on the basis of variation in size and colouration: *P*. *c*. *adustus* from northern Qld, *P*. *c*. *cinereus* from NSW, and *P*. *c*. *victor* from Vic. However, the geographical boundaries of these subspecies were poorly defined and therefore became associated with state political boundaries [17]. Although populations at the extremes of the distribution appear morphologically and genetically differentiated, this variation appears clinal and is insufficient to support subspecies classification [12,14,16,18].

As an internationally recognised wildlife icon, the koala presents complex and challenging management issues. The conundrum arises due to the differing impacts of threatening processes and previous management across their range [19]. Throughout most of their range koala populations are subject to several threatening processes including historical and ongoing habitat loss and fragmentation (due to agriculture and urbanisation) [8], climate change and drought [20–22], infectious diseases, notably chlamydia and Koala Retrovirus [23–26], and the effects of increased conflicts with humans [12,27,28]. At European settlement, koalas were not abundant but increased in numbers with reduced indigenous hunting [8]. Subsequent hunting of koalas in the 1900s to support international fur trade and extensive habitat loss resulted in declines across the range and the extirpation of many populations in the southern parts of the range by the 1930s [8,11]. As a result of substantial regional declines in Qld, NSW and the Australian Capital Territory koalas are listed as ‘vulnerable’ under the *Environmental Protection and Biodiversity Conservation Act 1999* [29]. In contrast, koalas occur at high densities in much of the southern parts of their range, leading to over-browsing [30] requiring population control measures to prevent starvation [8,31–33]. Koala management and conservation is often further complicated by jurisdictional issues between Australian State and Commonwealth laws. Thus, in order to effectively manage koalas at a national level, an holistic approach which incorporates information from a range of geographic scales and considers each area in a wider distributional context is required.

A series of studies have previously assessed the population genetics of koalas at a range of scales [18,34–49]. Together these studies have revealed information on dispersal, phylogeography, population history and genetic structure, but many have focused at local or regional scales, and few have attempted to place local populations into the broader national context. While the initial ground-breaking distribution-wide phylogeographic study by Houlden et al. [18] provided a strong basis for future studies, a subsequent lack of standard nomenclature and cross referencing for mitochondrial DNA (mtDNA) *Control Region* (*CR*) haplotypes resulted in difficulty placing subsequent smaller-scale studies into an overall national framework: a context which could have been used to effectively inform koala conservation and management.

This study revisits the question of koala phylogeography, examining the largest dataset gathered to date, including samples from a range of time periods (1870s to 2015) to provide information on the biogeographic patterns in eastern Australia and provide a robust framework for koala management and conservation. Specifically we aim to use mtDNA *CR* sequence data to investigate relationships among koala populations nationally, the impact of known biogeographic barriers, the extent of female mediated gene flow and signatures of demographic changes across the range. In addition, we aim to consolidate existing *CR* data to establish a coherent dataset which will enhance future studies and management decisions by providing a framework to enable local information to be considered in the context of the entire koala distribution.

## Materials and Methods

### Sampling

Tissue samples were obtained from 662 wild koalas from throughout the distribution (Fig 1, Table 1). Koala samples were obtained from researchers, consultants, as part of veterinary care at the Port Macquarie Koala Hospital and Australia Zoo Wildlife Hospital, and from the Australian Museum Tissue Collection. Sample collection was performed in accordance with methods approved by the Australian Museum Animal Ethics Committee (Permit Numbers: 11–03, 15–05). Australian Museum registration numbers are provided in S1 Table. Tissues samples were stored in 70–100% ethanol, RNA later or frozen at -20°C until DNA extraction.

**Table 1:** Mitochondrial diversity in the 20 sampled sites (n >7) across the range of koala, showing the number of haplotypes identified, haplotypic (*h*) and nucleotide (π) diversity and SD.

Site number^1^	Site location	Sample size	# haplotypes	Haplotypic diversity (*h)*	Nucleotide diversity (π)
1	Whitsunday/ Mackay, Qld	8	4	0.750 (±0.139)	0.0060 (±0.0037)
2	Blair Athol, Qld	10	2	0.200 (±0.154)	0.0028 (±0.0019)
3	Clermont, Qld	38	5	0.371 (±0.095)	0.0012 (±0.0011)
4	Maryborough, Qld	11	1	-	-
5	Redlands, Qld	7	2	0.476 (±0.171)	0.0017 (±0.0013)
6	Coomera, Qld	21	1	-	-
7	Tyagarah, NSW	17	1	-	-
8	Ballina, NSW	37	2	0.074 (±0.067)	0.001 (±0.0008)
9	Iluka, NSW	7	1	-	-
10	Pine Creek, NSW	50	1	-	-
11	Port Macquarie, NSW	142	3	0.450 (±0.031)	0.0011 (±0.0001)
12	Maitland, NSW	7	1	-	-
13	Campbelltown, NSW	24	4	0.663 (±0.060)	0.0023 (±0.0015)
14	Strzelecki/ East Gippsland, Vic	33	3	0.119 (±0.076)	0.0003 (±0.0042)
15	French Island, Vic	19	1	-	-
16	Cape Otway, Vic	14	1	-	-
17	Bessiebelle, Vic	33	2	0.061 (±0.052)	0.0001 (±0.0002)
18	Mt. Lofty Ranges, SA	30	6	0.662 (±0.058)	0.0029 (±0.0018)
19	Eyre Peninsula, SA	19	1	-	-
20	Kangaroo Island, SA	26	3	0.227 (±0.106)	0.0013 (±0.0009)
	Overall*	**662**	**36**	**0.840 (**±**0.079)**	**0.0073 (**±**0.0038)**

^1^ Site numbers refer to locations on Fig 1.

* All sampled contemporary koalas were included

### DNA extraction and mtDNA amplification

Genomic DNA was extracted using Qiagen DNeasy Blood and Tissue kit (Qiagen GmbH, Hilden, Germany) following standard protocols or according to a high salt method [50]. An ~850 base pair (bp) fragment of Domain 1 of the mtDNA *CR* (from the tRNA proline to the end of the central conserved region) was amplified using the marsupial-specific primers L15999M and H16498M [51]. PCRs were carried out in 25 μl reactions using 100–500 ng of genomic DNA, 1 x Reaction Buffer (Bioline My taq Red Reagent Buffer; Bioline, Australia), 2 pmol primers and Bioline My Taq Red DNA polymerase (0.5 unit). Negative controls were included in each PCR. Thermocycling was performed on an Eppendorf Mastercycler EpS (Eppendorf, Hamburg, Germany) under the following conditions; initial denaturation (94°C for 2 min); 38–45 cycles of denaturation (94°C for 20 s); annealing (60°C for 40 s) and extension (72°C for 50 s) followed by a final extension (5 min at 72°C). PCR products were cleaned using ExoSap-IT^©^ (USB Corporation, Cleveland, Ohio, USA). Sequencing was resolved on an AB 3730xl Sequencer at AGRF Sydney.

Additionally, sequence data for the mtDNA *CR* was obtained from five museum specimens (GenBank KJ530551 to KJ530556; [38,52]). These individuals dated from 1870 to 1938 and the dried museum skin specimens were provided by the Bohusläns Museum, Gӧteborg Museum, Museum of Comparative Zoology at Harvard University, Queensland Museum and Stockholm Museum. All work was carried out in a laboratory dedicated to ancient DNA experiments (in the Leibniz Institute for Zoo and Wildlife Research) to avoid the risk of contamination with modern DNA. The DNA extraction, library and bait preparation and hybridisation procedures, and bioinformatics analyses for these samples are described in Tsangaras et al. [38] and Tsangaras et al. [52]. Hybridization capture PCR product baits were generated from modern koala genomic DNA (SN265; KJ530552.1) with *CR* primers (PCI-CR-NF:5′-CATCAACACCCAAAGCTGAT-3′ and PCI-CR-NR: 5′-TTCTAGGTACGTCCGCAATCT-3′). Subsequent library and bait preparation, and hybridisation capture are described in [52], with sequencing on an Illumina MiSeq platform at University of Copenhagen National High-throughput DNA Sequencing Center. Bioinformatics procedures are described in [52] with consensus sequences for all samples generated using Geneious v 6.1.8 [53].

### Mitochondrial DNA analysis

Sequences were checked and edited with reference to chromatograms using Sequencher v 5.3.2 (Gene Codes Corporation, Ann Arbor, MI, USA). Any individuals with ambiguous sequence data or unique singleton haplotypes (i.e. it was the only individual possessing that sequence) were re-amplified and sequenced for verification. Unique haplotype sequences were lodged with GenBank under accession numbers KX618862 –KX618876).

In addition to the unique sequences generated in this study, we obtained existing published koala haplotypes available on GenBank (accession numbers AJ005846—AJ005863; KJ530551—KJ530556; KC505325; GQ851933—GQ851940; AJ012057—AJ012064; KF745869—KF745875). Since a number of koala mtDNA sequences of varying sizes exist, we compared all the existing haplotypes, identifying potential duplicates (i.e. identical sequences based on accessioned data). All haplotypes were then assigned standardised names as described in Table 2. All existing and novel sequences were then aligned using the CLUSTAL X algorithm implemented in MEGA 6 [54]. The most appropriate model of evolution was determined using jMODELTEST version 1.1 [55,56] using the Akaike Information Criterion (AIC).

**Table 2:** List of the 53 unique mitochondrial DNA Control Region haplotypes found in koala.

Standardised name	Previously published as	Genbank Accession number	Referenced in	Locations reported in this study
Pc1		KX618862.1	This study	k
Pc2	A-6^1^	KF745874.1	This study, [57]	10, 11, 16, p, q, t,
Pc3		KX618865.1	This study	11, k, p
Pc4	H10	AJ005855.1	This study, [18]	12, l, m, n, o
Pc5		KX618871.1	This study	k
Pc6	A-17^1^	KF745869.1	This study, [57]	j
Pc7	H5	AJ005850.1	This study, [18,41,45]	6, 7, 8, 9, e, f, h, l, t
	Q1^1^	AJ012057.1		
	B^1^^,^^3^			
Pc8		KX618875.1	This study	10
Pc9		KX618876.1	This study	p
Pc10		KX618863.1	This study	q
Pc11		KX618864.1	This study	p
Pc12	H1	AJ005846.1	[18]	
Pc13	H2	AJ005847.1	This study, [18,38,41,45,52]	1, 8, b, d, g
	Q8^1^	AJ012064.1		
	D^3^			
	Pci-maex1738^2^ (K5)	KJ530551.1		
Pc14	H3	AJ005848.1	This study, [18,41,45]	i
	Q4^1^	AJ012060.1		
	E^3^			
Pc15	H4	AJ005849.1	This study, [18,41,45]	5
	Q2^1^	AJ012058.1		
	A^1^^,^^3^			
Pc16	H6	AJ005851.1	This study, [18]	m, n
Pc17	H7	AJ005852.1	This study, [18]	18, 20
Pc18	H8	AJ005853.1	[18]	NS
Pc19	H9	AJ005854.1	This study, [18]	13, 14, 18, m, o
Pc20	H11	AJ005856.1	This study, [18]	18
Pc21	H12	AJ005857.1	This study, [18]	13, 18, s
Pc22	H13	AJ005858.1	This study, [18]	18
Pc23	H14	AJ005859.1	This study, [18]	NS
Pc24	H15	AJ005860.1	[18]	NS
Pc25	H16	AJ005861.1	This study, [18]	14, 20
Pc26	H17	AJ005862.1	[18]	NS
Pc27	H18	AJ005863.1	This study, [18]	11, 13, 14, 15, 17, 18, 19, 20, s
	B-9^1^	KF745870.1	[57]	
Pc28	Q7^1^	AJ012063.1	This study, [38,41,45,52,57]	4
	O^3^			
	B-12^1^	KF745873.1		
	Pci-um3435^2^ (K3)	KJ530553.1		
Pc29	A-15^1^	KF745872.1	[57]	NS
Pc30	B-18^1^	KF745871.1	[57]	NS
Pc31	B-4^1^	KF745875.1	This study, [38,52,57]	1, 2, 3
	Pci-QMJ6480^2^ (K5)	KJ530554.1		
Pc32	C^1^	GQ851933.1	[45]	NS
Pc33	G^1^	GQ851934.1	[45]	NS
Pc34	H^1^	GQ851935.1	[45]	NS
Pc35	I^1^	GQ851936.1	[45]	NS
Pc36	J^1^	GQ851937.1	[45]	NS
Pc37	K^1^	GQ851938.1	[45]	NS
Pc38	M^1^	GQ851939.1	[38,45,52]	NS
	Pci-582119^2^ (K4)	KJ530556.1		
Pc39	N^1^	GQ851940.1	[45]	NS
Pc40	Q3^1^	AJ012059.1	[41,45]	NS
	F^1,3^			
Pc41	Q5^1^	AJ012061.1	[41]	NS
Pc42	Q6^1^	AJ012062.1	This study, [41,45]	5, e
	L^3^			
Pc43	St. Bees Island^1^	KC505325.1	This study, [34]	3
Pc44		KX618866.1	This study	o
Pc45		KX618867.1	This study	u
Pc46	Pci-SN265	KJ530552.1	This study, [38,52]	1, 2, 3
Pc47		KX618868.1	This study	g
Pc48		KX618869.1	This study	17
Pc49		KX618870.1	This study	c
Pc50		KX618872.1	This study	3
Pc51		KX618873.1	This study	1, a, b
Pc52		KX618874.1	This study	3
Pc53	Pci-MCZ8574^2^ (K4)	KJ530555.1	[38,52]	NS

The standardised names (Pc1-53) are shown against names of matching sequences from Genbank and used in the literature.

^1^ short (~600 bp) sequences.

^2^ haplotypes obtained from historic specimens. The haplotype name used by Tsangaras [38] for these samples is shown in brackets.

^3^ haplotypes that had been matched to previously reported haplotypes, but were named using the respective studies’ nomenclature, rather than that associated with the accession number. For locations abbreviations refer to Fig 1

#### Phylogenetic analyses

Phylogenetic relationships amongst all 53 unique haplotypes (i.e. novel haplotypes reported here and those from previous studies) were estimated using Maximum Likelihood (ML) and Bayesian Inference (BI). ML analyses were implemented in GARLI 2.1 Web service [58]. The best ML tree was estimated using an adaptive search method with 1000 replicates to determine the optimal topology with 0.95 probability. The gamma distribution and proportion of invariant sites were estimated in GARLI. Support for the branching topology was evaluated with 1000 bootstrap replicates. BI analyses were calculated in BEAST v1.8.3. [59,60] using a Metropolis-coupled Markov chain Monte Carlo sampling approach run for 10^7^ iterations, sampling every 1000^th^. The most appropriate model of evolution available was selected, the Hasegawa–Kishino–Yano model (HKY; [61]), based on the results of jMODELTEST. Three independent replicates were conducted and inspected for consistency to check for local optima in TRACER. In addition a range of priors (including the default settings) were run and the robustness of the data assessed in TRACER (S1 File). Mitochondrial DNA *CR* sequence from the common wombat (*Vombatus ursinus*; NC_003322.1), the closest relative of the koala [62], was used as an outgroup in all analyses. The maximum credibility lineage tree and posterior probabilities were calculated in TREE ANNOTATOR, with the first 1000 trees discarded as burn-ins. A measure of within-group differences (D_A_) was calculated between all identified lineages within the tree using MEGA.

A haplotype network was created including all 53 unique koala mtDNA *CR* haplotypes using the TCS procedure [63], which uses the probability of parsimony calculated for pairwise comparisons to create the network [64], implemented in PopART (http://popart.otago.ac.nz). Information on the frequency of haplotypes was only obtained from the sampling in the present study. Indels were coded as a single mutation. Two networks were generated: one including all 53 sequences with missing data masked and another including only sequences with >850 bp. To combine all the available information we manually compared and combined the two networks. We took a conservative approach to the number of mutations in short sequences, assuming they were identical to longer sequences where data was missing.

#### Mitochondrial DNA diversity and partitioning

For sites sampled in this study where at least 7 individuals were sampled, the levels of mtDNA *CR* diversity and differentiation were assessed. Haplotypic diversity (h), nucleotide diversity (π) and pairwise differentiation (Φ_ST_) amongst populations were estimated using ARLEQUIN v3.5.2.2 [65]. Deviations from neutrality were examined for each of these 20 sampling localities, and overall using Fu’s F_S_ [66] and Tajima’s D-statistic [67].

Partitioning of mtDNA diversity was assessed using an Analysis of Molecular Variance (AMOVA), implemented in ARLEQUIN. Partitioning was examined (i) within vs between populations across the range to determine general patterns of differentiation, and (ii) between populations from previously described subspecies (corresponding to Qld, NSW and Vic/SA) to determine if subspecies correspond to genetic partitioning and (iii) the same as (ii) but with all known reintroduced populations removed from the analysis, as translocated populations may disrupt natural levels of differentiation. A Spatial Analysis of Molecular Variance (SAMOVA), implemented in SAMOVA v2 was used to define homogenous groups [68]. This analysis partitions the populations into a specified number of clusters, maximising the variation between clusters (F_CT_). The analysis was run with and without location data. To determine the most appropriate grouping for our dataset we estimated F_CT_ for up to 20 populations (i.e. the number of sampled locations).

The influence of geographic distance on the extent of differentiation (Φ_ST_) was also assessed using Isolation By Distance, Web Service (IBDWS) version 3.23 [69]. The natural logarithm of Φ_ST_/(1- Φ_ST_) [70] and geographic distance was used due to the level of variance in the dataset and the significance of this relationship was estimated using a Mantel’s test, with 10000 permutations. This was tested using sites containing >7 individuals (see Table 1) and repeated using all sampled sites containing more than one individual.

#### Population history and demography

Mismatch analysis was performed in ARLEQUIN to test for historical demographic expansion events in our sampled populations [65]. We tested for historical expansion events in the species overall (i.e. all samples as a single population), and for each of the geographically defined lineage identified by phylogenetic analyses. Since koalas in the northern and southern regions of the range have different population histories [9,19], we tested for demographic events between populations where koalas have been extensively translocated (Vic/SA) and primarily natural populations (NSW/Qld). Concordance between these models and the empirical data was assessed by means of a least-squares approach [71]. To further assess demographic changes in koala populations Bayesian skyline plots (BSP) were generated in BEAST for each of the mtDNA *CR* lineages and the overall dataset. The HKY model of substitution (the most appropriate available based on jMODELTEST) was selected and a substitution rate of 15% per million years was selected, which has previously been applied to several macropod marsupials [5,6]. There are currently no suitable calibration points/fossils to calibrate a molecular clock for the koala. The analysis was run for 10^7^ generations, sampling every 10000^th^ generation. The results were visualised in TRACER and calibrated for generation time (approximately 7 years; [72]).

## Results

A total of 36 unique mtDNA *CR* haplotypes were identified in the 662 contemporary koalas sampled in this study. When compared to haplotypes previously accessioned to GenBank, 15 haplotypes were found to be novel and 21 had previously been reported (Table 2). These 36 haplotypes were defined by 46 variable sites, of which 26 were parsimony informative.

Searches of existing mtDNA *CR* data for koalas revealed 48 haplotypes have been published in the literature and accessioned into GenBank (Table 2). These sequences vary in size and sections of the *CR* but fall into approximately 600 bp fragments and 850 bp fragments, where the larger fragment entirely overlaps the smaller fragments. We found that only 36 of these 48 haplotypes represent unique sequences. In order to align existing datasets a summary of these haplotypes, including matching haplotypes and our proposed standardised haplotype nomenclature is shown in Table 2. The standardised haplotype names outlined in Table 2 are referred to throughout the rest of this paper.

### Phylogenetic analyses

**Fig 2:**
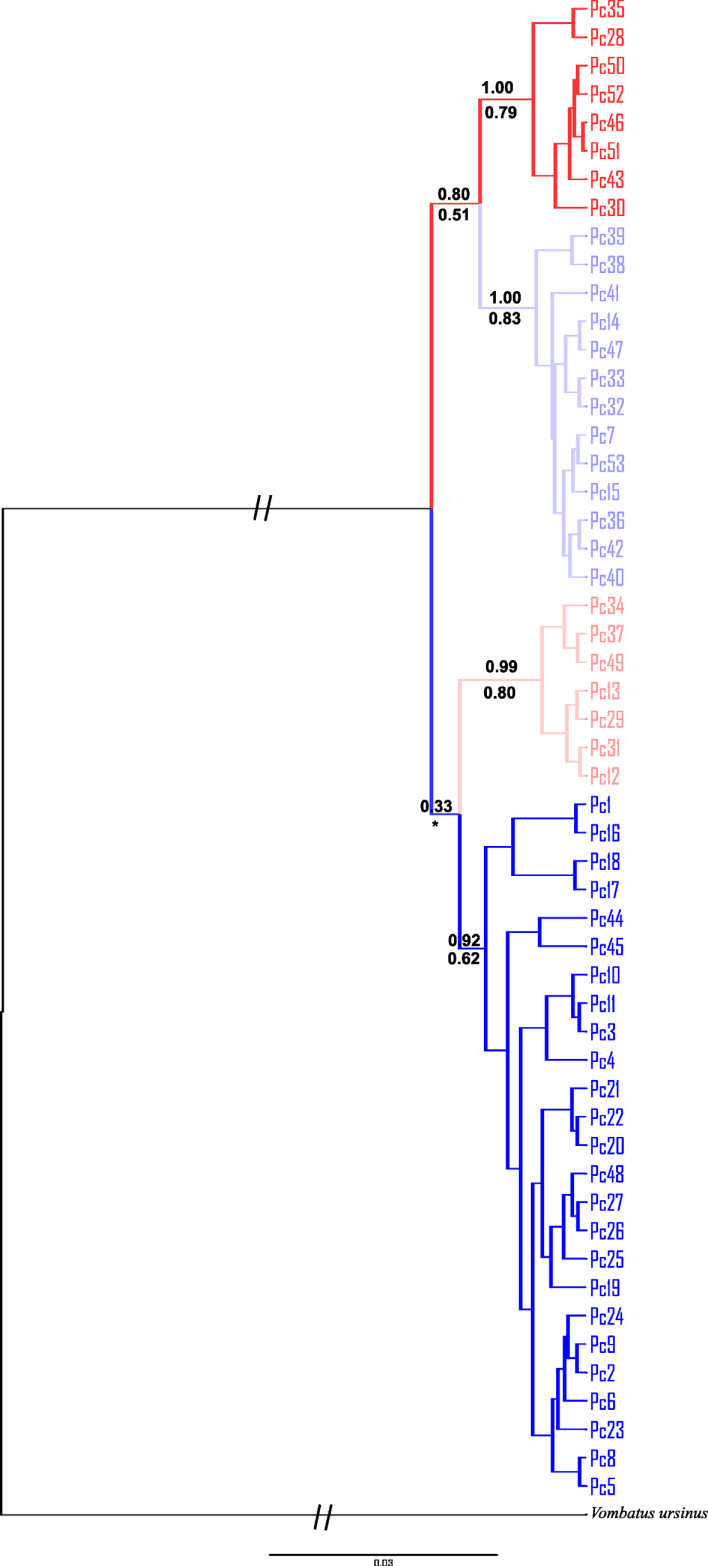
Phylogenetic tree showing the relationship between 53 mitochondrial DNA *Control Region* koala haplotypes. The maximum credibility clade tree based on Bayesian Inference (BI) is shown, using the common wombat (*Vombatus ursinus*; NC_003322.1) as an outgroup. The posterior probabilities of the main branches are shown, with BI value above the line and ML below. *indicates branches not supported by ML. Red = northern lineage 1, light red = northern lineage 2, purple = central lineage, blue = southern lineage.

**Fig 3:**
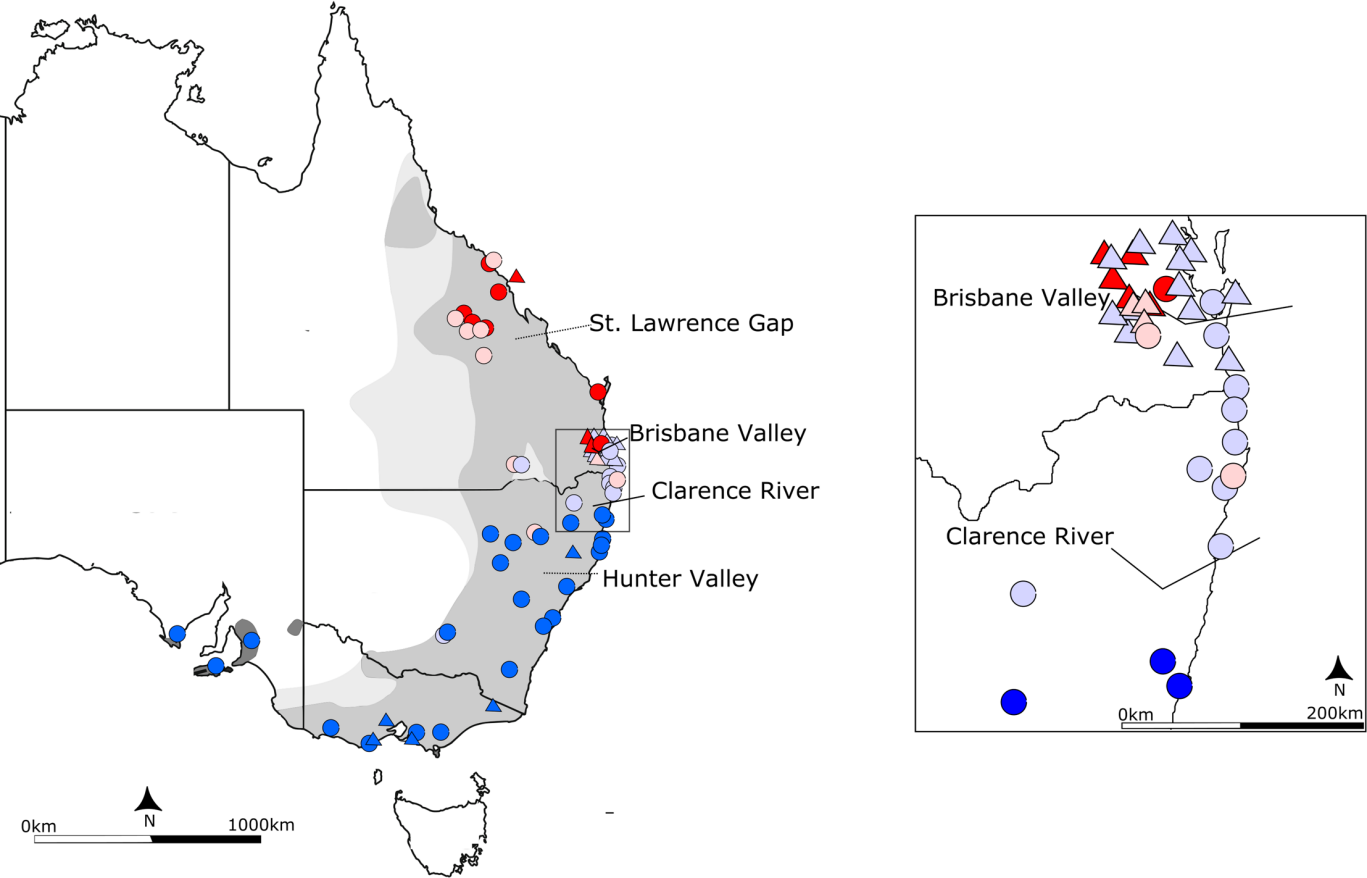
Geographical distribution of the koala, *Phascolarctos cinereus*, showing the locations of sampled mitochondrial DNA *Control Region* lineages. The current distribution is shown in grey, with the historical range in light grey and introduced populations in dark grey. Sampling locations for this study are shown as circles with triangles representing sites only sampled by previous studies. The identified mitochondrial DNA *Control Region* lineages are represented by colours (northern lineage 1: red; northern lineage 2: light red; central lineage: purple; southern lineage: blue). Biogeographic barriers that appear to have impacted on koalas, Brisbane Valley (BVB) and Clarence River (CRB) are shown as solid lines; other putative barriers are shown as dashed lines (St. Lawrence Gap and Hunter Valley). The inset shows the distribution of sampled haplotypes around the two proposed biogeographic barriers, BVB and CRB.

**Fig 4:**
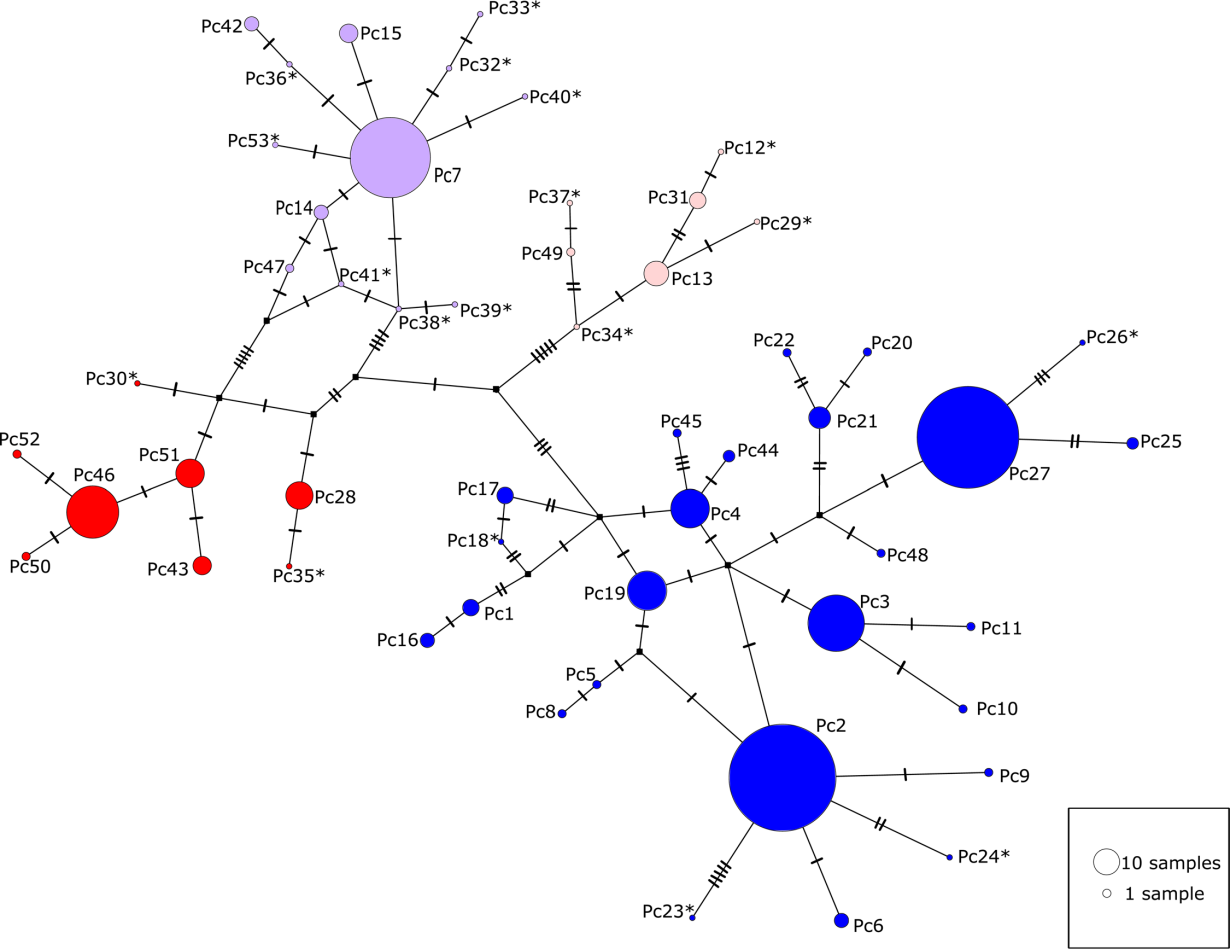
Haplotype network for koala mitochondrial DNA *Control Region* haplotypes. The size of the circles are proportional to the number of individuals represented. * denotes haplotypes obtained from GenBank (and not detected in our study), which are represented by a single individual. Squares represent unsampled hypothesised haplotypes. Crosses on connecting lines indicate the number of mutational steps between haplotypes. Red = northern lineage 1, light red = northern lineage 2, purple = central lineage, blue = southern lineage.

The topology of the phylogenetic trees generated using BI and ML were similar. The BI maximum credibility lineage tree is shown in Fig 2. Four well supported but shallowly diverged lineages were present, with each mostly confined to discrete geographic areas. Lineages 1 and 2 were found north of Brisbane, Lineage 3 between Brisbane (i.e. Redlands; site 5) and Iluka (site 9), and Lineage 4 haplotypes occurred south of Iluka, although there is evidence of admixture across these boundaries (Figs 3 and 4). There was no consistent evidence of further substructure in the trees. The haplotype network (Fig 4) illustrates how shallow the divergences are amongst the four lineages, which ranged between 1–1.6%.

### Mitochondrial DNA diversity and partitioning

Overall haplotype diversity of the koalas sampled in this study was 0.84 (±0.08) but varied substantially from 0.12 to 0.75 across the sites sampled. Nucleotide diversity was 0.73% across all koalas sampled, and again ranged widely among the sampling sites from no variation (i.e. a single haplotype) to 0.60% within-sites (Table 1). The results from some populations should be treated with caution due to the limited number of samples. A comparison between the 850 bp fragments of the five historic samples and contemporary samples revealed that only one of these haplotypes was not represented in the contemporary samples. Significant deviations from neutrality were detected in each of the lineages and overall for Fu’s F_S_, but Tajima’s D values were not significant. This suggests the deviation from neutrality is relatively weak since Fu’s F is more sensitive than Tajima’s D to deviations from neutrality [73].

The results of the AMOVA revealed that genetic diversity was partitioned between sampling sites (88.84% of the variation) rather than within sites (11.16%). There was evidence of significant partitioning of genetic diversity between the previously described subspecies (both including and excluding introduced populations) but only moderate amounts of genetic diversity were partitioned between the ‘subspecies’ (35–43%), relative to between sites within (50–54%). The SAMOVA analysis identified three as the optimal number of groups. Regardless of incorporating geographic data the SAMOVA separated populations between Maryborough (site 4) and Redlands (site 5), and between Iluka (site 9) and Pine Creek (site 10).

Pairwise comparisons of population differentiation ranged from 0 to 1, and significant differences occurred between most sites (Table 3). The exceptions were either geographically proximate (e.g. Coomera (site 6) and Ballina (site 8)) or occurred in the southern parts of the range where translocations have occurred (e.g. French Island (site 15) and Eyre Peninsula (site 19)). There were also instances of geographically proximate sites being genetically divergent (e.g. Pine Creek (site 10) and Iluka (site 9)). There was no significant relationship between genetic and geographic distances when sites containing seven or more individuals were included (p = 0.98). There was a significant positive relationship when all the sampling sites (with n > 1) were included (p = 0.01), but this explained only 13% of the variation.

**Table 3:** Levels of differentiation (ΦST) between samples localities (n > 7) of koalas.

1. Whitsunday/ Mackay	**-**																			
2. Blair Athol	0.09	**-**																		
3. Clermont	**0.21**	0.00	**-**																	
4. Maryborough	**0.57**	**0.78**	**0.75**	**-**																
5. Redlands	**0.72**	**0.84**	**0.87**	**0.94**	**-**															
6. Coomera	**0.87**	**0.94**	**0.91**	**1.00**	**0.66**	**-**														
7. Tyagarah	**0.84**	**0.93**	**0.90**	**1.00**	**0.62**	0.00	-													
8. Ballina	**0.81**	**0.89**	**0.88**	**0.93**	**0.39**	0.00	0.00	-												
9. Iluka	**0.75**	**0.88**	**0.88**	**1.00**	**0.44**	0.00	0.00	0.00	**-**											
10. Pine Creek	**0.92**	**0.96**	**0.94**	**1.00**	**0.98**	**1.00**	**1.00**	**0.97**	**1.00**	**-**										
11. Port Macquarie	**0.89**	**0.92**	**0.91**	**0.92**	**0.93**	**0.94**	**0.94**	**0.93**	**0.93**	**0.20**	**-**									
12. Maitland	**0.74**	**0.88**	**0.88**	**1.00**	**0.94**	**1.00**	**1.00**	**0.93**	**1.00**	**1.00**	**0.63**	**-**								
13. Campbelltown	**0.80**	**0.88**	**0.88**	**0.92**	**0.91**	**0.95**	**0.95**	**0.92**	**0.93**	**0.75**	**0.58**	**0.55**	**-**							
14. Strzelecki/ East Gippsland	**0.89**	**0.94**	**0.92**	**0.98**	**0.97**	**0.99**	**0.99**	**0.96**	**0.99**	**0.92**	**0.60**	**0.87**	**0.19**	**-**						
15. French Island	**0.86**	**0.94**	**0.91**	**1.00**	**0.97**	**1.00**	**1.00**	**0.96**	**1.00**	**1.00**	**0.59**	**1.00**	**0.20**	0.00	-					
16. Cape Otway	**0.85**	**0.93**	**0.91**	**1.00**	**0.97**	**1.00**	**1.00**	**0.96**	**1.00**	0.00	**0.16**	**1.00**	**0.65**	**0.89**	**1.00**	**-**				
17. Bessiebelle	**0.90**	**0.95**	**0.93**	**0.99**	**0.98**	**1.00**	**1.00**	**0.96**	**1.00**	**0.97**	**0.61**	**0.95**	**0.25**	0.00	0.00	**0.96**	**-**			
18. Mt. Lofty Ranges	**0.78**	**0.85**	**0.86**	**0.89**	**0.88**	**0.92**	**0.92**	**0.90**	**0.90**	**0.65**	**0.58**	**0.46**	0.00	**0.19**	**0.18**	**0.55**	**0.22**	**-**		
19. Eyre Peninsula	**0.86**	**0.94**	**0.91**	**1.00**	**0.97**	**1.00**	**1.00**	**0.96**	**1.00**	**1.00**	**0.59**	**1.00**	**0.20**	0.00	0.00	**1.00**	0.00	**0.18**	**-**	
20. Kangaroo Island	**0.83**	**0.90**	**0.89**	**0.94**	**0.93**	**0.96**	**0.96**	**0.93**	**0.95**	**0.76**	**0.56**	**0.57**	**0.09**	0.01	0.02	**0.67**	0.04	**0.09**	0.02	**-**
	1.	2.	3.	4.	5.	6.	7.	8.	9.	10.	11.	12.	13.	14.	15.	16.	17.	18.	19.	20.

Bold denotes significant values at 5% level

### Population history and demography

**Fig 5:**
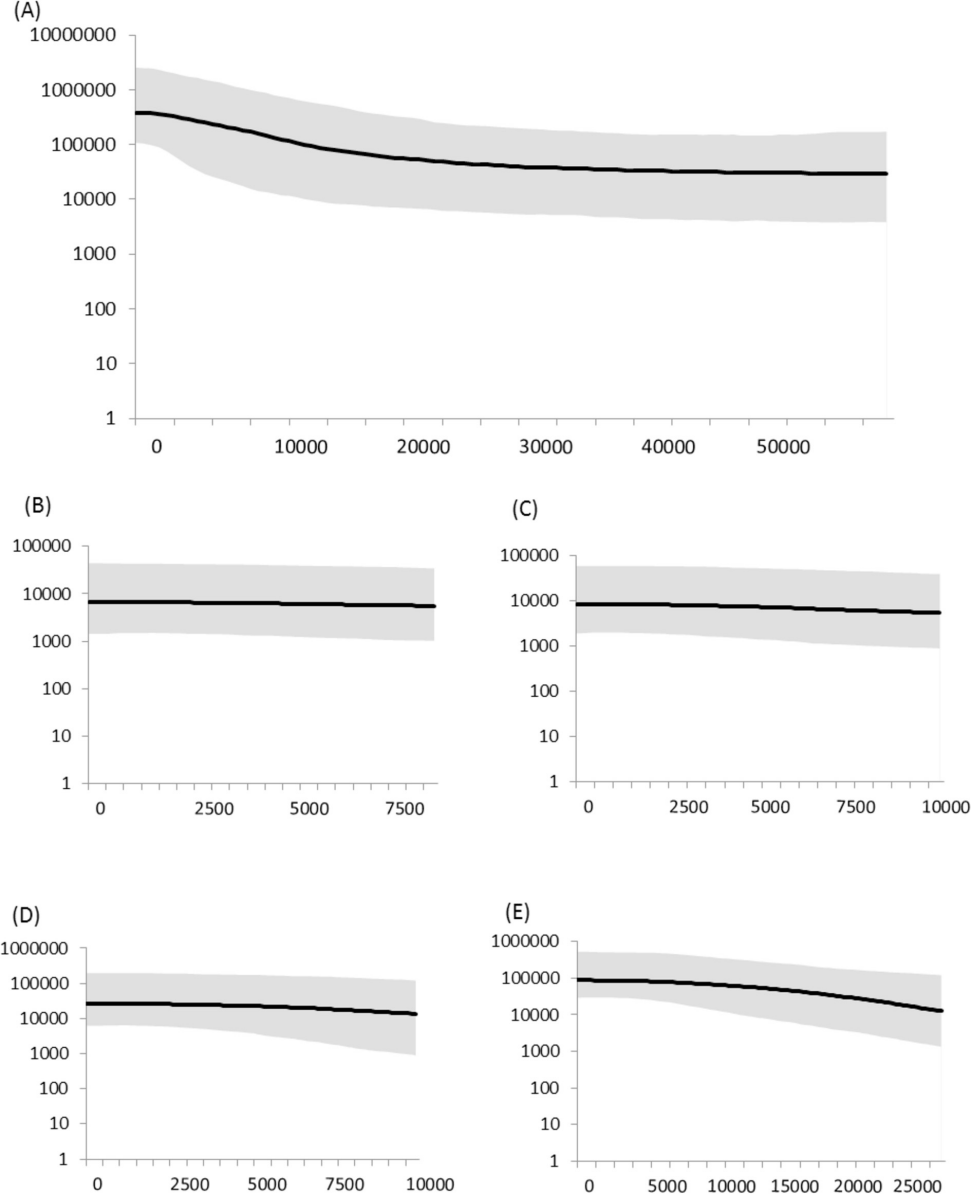
Bayesian skyline plots of the effective population size over time for the koala. (A) overall, (B) northern lineage 1 (C) northern lineage 2, (D) central lineage and (E) southern lineage. Median estimates are shown as solid lines and shading represents the 95% highest posterior density intervals.

The results of the mismatch analysis were consistent with a model of spatial expansion in all of the *a priori* groups tested, although the grouping of NSW and Qld haplotypes and the southern lineage failed to converge after 2000 steps. Models of demographic expansion could not be rejected as the non-linear least squares algorithm failed to converge after 2000 steps for all *a priori* groupings. BSP showed each lineage appeared relatively stable over time, except for the southern lineage, which exhibited an increase in population size (Fig 5). When assessed overall, BSP suggests koala numbers began increasing approximately 20000 years ago, and stabilised at the current size approximately 3000 years ago.

## Discussion

### Phylogeography and genetic structure

Our study represents the largest and most extensive sample set yet used to investigate the phylogeography of the koala throughout its range, and suggests the koala has a more complex biogeographic history than previously considered. The four shallow genetic lineages identified here do not correspond to previously described sub-species, and it is now apparent that morphological variation within the koala represents a cline reflecting gradients in environmental conditions across the distribution [12,16]. In a previous phylogeographic study, Houlden et al. [18] found a similar pattern of genetic divergence, noting the presence of ‘three clusters’ in their phylogenetic analyses, which correspond to three of the four identified in the present study. However, further interpretation was hindered by their limited sampling (particularly from northern regions) and the presence of haplotypes from multiple lineages at a single site. Here, the more extensive sampling (and more powerful phylogenetic analyses) revealed additional fine scale details and the ability to delineate these lineages. The extent of mixing suggests these biogeographic features are no longer significant barriers to movement, and other factors such as habitat availability are likely to have a greater impact on contemporary gene flow as evidenced by several recent population genetics studies (e.g. [37,41,44]). For example, Dennison et al. [49] demonstrated dispersal throughout the region where the central (3) and southern (4) lineages adjoin. Thus, these results strengthen the conclusions of Houlden et al. [18] that the koala represents a single species (or Evolutionary Significant Unit (ESU); *sensu* Moritz [74]) with no sub-specific separation.

Two biogeographic barriers, the BVB and CRB, previously identified in other eastern Australian species, have impacted the movement of koalas. The BVB appears to separate the two northern lineages (Lineages 1 and 2) from the central lineage (Lineage 3). This biogeographic barrier has previously been reported as impacting the phylogeography of a range of species including mammals [75], amphibians [76], reptiles [77] and invertebrates [78]. Detailed analysis of koalas in this region by Lee et al. [45] however, indicated haplotypes belonging to the central lineage (Lineage 3) occur along the coast north of the BVB (e.g. Brisbane area). This may be the result of movement following a reduction in the effectiveness of Pleistocene barriers, or suggest a role for the D’Aguilar Range, which runs roughly north–south and separates areas containing central lineage haplotypes (e.g. Brisbane area) from those with northern haplotypes (e.g. Brookfield (site d)) [45]. Further south, the central lineage (Lineage 3) and the southern lineage (Lineage 4) are separated by the CRB. Like the BVB this barrier no longer presents a substantial obstruction to movement as there is evidence of mixing of the two lineages over a large geographic area both in mtDNA (this study) and evidence of contemporary gene flow [49]. The CRB is apparent in several marsupials including long-nosed potoroos (*Potorous tridactylus* [6]), brush-tailed rock wallaby (*Petrogale pencillata* [79]) and Hastings River mouse (*Pseudomys oralis* [80]) as well as several reptile species [81]. Finally, our phylogenetic analyses indicated the presence of two lineages in the northern parts of the range, but there was extensive mixing across a broad geographic area and it was not possible to elucidate any potential barriers using our data. To date the genetics of the koalas in this region have been poorly investigated and further sampling in this area will be required to identify if any biogeographic barriers previously identified in other species have impacted on the koala in this region (e.g. SLG; [75,82,83]).

There is no evidence of barriers further south, such as the HVB impacting on phylogeographic patterns in koalas, reflecting the idiosyncratic nature of many Pleistocene barriers [6,79]. It is possible that the widespread translocation of koalas throughout most of the southern parts of the range (i.e. Vic) has masked any potential phylogeographic patterns in this region. The spread of haplotypes throughout Vic and SA reflects koalas’ more recent history of documented translocations, and suggests some potentially undocumented translocations. For example, koalas at Eyre Peninsula (site 19), Kangaroo Island (site 20) and Bessiebelle (site 17) are all similar to French Island (site 15) which, along with Phillip Island, was the reported source population [9]. In contrast, the Mt. Lofty koalas exhibit high levels of diversity, among the highest of the southern populations, consistent with their founding individuals being obtained from a wider area including NSW and Qld [9]. It has been proposed that the genetic diversity of koalas in the Strzelecki and south Gippsland areas may not have been as heavily impacted by translocations as other areas [18,39,46]. However, the levels of diversity present are still comparatively low. Interestingly, koalas from Cape Otway possessed a single haplotype not found in other Victorian sites, but that we documented in northern and western NSW locations. This is inconsistent with documented translocations from French and Phillip Islands to this general area [9]. It is likely this is indicative of undocumented translocations, but further work would be required to distinguish between remnant haplotypes and undocumented translocations.

Overall, the spread of haplotypes and lack of substructure within lineages suggests koalas have maintained effective (female) genetic connectivity historically (i.e. over evolutionary time). However, significant differentiation and partitioning of mtDNA *CR* diversity among sites is indicative of limited female movement on shorter timescales (i.e. ecological time). These results are consistent with previous regional and local population genetic studies of koalas and suggests that differentiation of koala populations is associated with contemporary (i.e. Post-European) barriers to dispersal [18,44,45,49]. Furthermore, ecological studies have suggested that koalas are capable of moving large distances [84,85]. In contrast to Qld and NSW, populations in Vic and SA exhibit low levels of divergence, which is consistent with previous genetic studies and the management history of koalas in these regions [9,18,39]. Together this information suggests that recent human-induced landscape changes and habitat loss coupled with founder and bottleneck effects are likely to have contributed to this pattern.

### Historic genetic diversity and demography

The levels of mtDNA diversity detected in koalas in this study are typical of arboreal species and those with specific habitat requirements [6]. The combination of habitat loss, disease and intesense hunting pressure to support an international fur trade resulted in dramatic declines in koala populations and the exitrpation of several southern populations by the 1930s [8,9,11]. The impact of such declines would be expected to be apparent in the genetics of koalas [47,66] and several studies have found evidence of genetic bottlenecks in some populations [39,47]. Sites in SA and Vic exhibited lower levels of diversity compared with those in Qld and NSW, which may be associated with reintroduction from a very limited source following extirpation [9,18]. However, we found no evidence of recent genetic bottlenecks or declines using mtDNA *CR* data; rather our BSP indicated koala populations have remained relatively stable or increased during the past 1000 years. Tsangaras et al. [38] found little evidence of the loss of diversity in mtDNA *CR* haplotypes when comparing contemporary samples to those from the late 1800s and early 1900s. Furthermore, our extensive sampling of contemporary koalas revealed that only one of these haplotypes appears restricted to historic specimens.

The BSP does however suggest a post-LGM expansion, particularly into the southern regions of the range (Fig 5). This pattern of expansion is consistent with bioclimatic models, which suggest koalas were largely restricted to several fragmented areas in northeastern NSW and southeastern Qld during the LGM [86]. It is also possible that this earlier expansion event has led to low levels of diversity in this region which hamper the detection of this most recent bottleneck (particularly when combined with the lower effective size of mtDNA) [42,87]. Much more work, particularly studies including samples from Pleistocene deposits will be required to elucidate patterns of mtDNA *CR* diversity loss in koalas.

### Aligning mtDNA datasets

Sequence variation in mtDNA *CR* has been widely employed to assess genetic diversity and phylogeography, and still forms the basis of management programmes for many species [88]. In koalas, mtDNA has been utilized at a range of scales, including local and distributional scales, using both contemporary and historic samples [18,38,41,45]. Sequence data is easily transferable between labs, and readily shared via online repositories (i.e. GenBank). However, to date different studies have employed different naming systems and there is some duplication of sequences (but with different names assigned by different authors), which can add to confusion about levels of diversity and structure. Table 2 matches new and existing haplotypes and integrates them into a single nomenclature. This will improve the integration of datasets, but it is essential that researchers adopt common nomenclature to ensure consistency and comparability in the future. We acknowledge that this is not straightforward where different length sequences are used. In the long term, the decreasing costs of Next Generation Sequencing (NGS) and use of whole mitogenome sequencing may reduce this problem but currently, targeted sequencing of mtDNA *CR* still forms the basis of many management strategies. We suggest researchers undertake simple measures such as ensuring only new unique sequences are named. For complex systems, it may be necessary for researchers to adopt strict nomenclature systems, such as those used in human genetics. One option is adopting the revised Cambridge Naming System (rCNS), where haplotype names are based on the SNPs which distinguish that haplotype from a reference sequence [89,90]. This minimises the length of names but also provides some information on the relationships among haplotypes. Adopting such conventions would require a shift in the approach of wildlife researchers to a more complex system, but one which may streamline phylogeographic studies, particularly where multiple local studies exist.

### Management and Conservation

Koala conservation is not straightforward, due to the varying impacts of threatening processes and jurisdictional issues [19]. Effective management requires information at a range of scales but ensuring local or regional level studies can be placed into the broader distributional context. To date, this has not been achieved for koalas. To address this issue we have: (1) Synthesised existing data for mtDNA *CR*, providing a standardised nomenclature and framework for management and future work (Table 2); and (2) Used the largest dataset to date to assess the phylogeography of koalas that can be used to inform management. Our results confirm that koalas should be managed as a single ESU, with no sub-specific separation. However, the presence of low levels of genetic divergence resulting from Pleistocene barriers and morphological variation, which most likely reflects environmental clines [14,16], should be taken into consideration in maximising the conservation of genetic diversity. These results also highlight the importance of nationally significant koala populations in southeastern Qld and northeastern NSW, as this area contains a high proportion of the mtDNA diversity present in koalas, but which is under threat from urbanisation. This is particularly important, given these results support previous inferences that movement and dispersal in koalas has been restricted by habitat fragmentation. This nationwide phylogeographic study provides a strong framework for fitting information from local studies into a national context.

## Supporting Information

S1 File BEAST input files. (ZIP)

S1 Table Sample information. (XLSX)

## References

[1] Byrne M. Evidence for multiple refugia at different time scales during Pleistocene climatic oscillations in southern Australia inferred from phylogeography. Quat Sci Rev. 2008;27: 2576–2585. 10.1016/j.quascirev.2008.08.032

[2] Byrne M, Yeates DK, Joseph L, Kearney M, Bowler J, Williams MAJ, et al Birth of a biome: insights into the assembly and maintenance of the Australian arid zone biota. Mol Ecol. 2008;17: 4398–417. 10.1111/j.1365-294X.2008.03899.x 18761619

[3] Byrne M, Steane DA, Joseph L, Yeates DK, Jordan GJ, Crayn D, et al Decline of a biome: evolution, contraction, fragmentation, extinction and invasion of the Australian mesic zone biota. J Biogeogr. 2011;38: 1635–1656. 10.1111/j.1365-2699.2011.02535.x

[4] Hewitt G. The genetic legacy of the Quaternary ice ages. Nature. 2000;405: 907–13. 10.1038/35016000 10879524

[5] Neaves LE, Zenger KR, Prince RIT, Eldridge MDB. Impact of Pleistocene aridity oscillations on the population history of a widespread, vagile Australian mammal, *Macropus fuliginosus*. J Biogeogr. 2012;39: 1545–1563.

[6] Frankham GJ, Handasyde KA, Eldridge MDB. Evolutionary and contemporary responses to habitat fragmentation detected in a mesic zone marsupial, the long-nosed potoroo (*Potorous tridactylus*) in south-eastern Australia. J Biogeogr. 2016;43: 653–665. 10.1111/jbi.12659

[7] Bryant LM, Krosch MN. Lines in the land: a review of evidence for eastern Australia’s major biogeographical barriers to closed forest taxa. Biol J Linn Soc. 2016; 10.1111/bij.12821

[8] Martin RW, Handasyde KA. The Koala: Natural History, Conservation and Management. UNSW Press; 1999.

[9] Menkhorst P. Hunted, marooned, re-introduced, contracepted: a history of Koala management in Victoria In: Lunney D, Munn A, Meikle W, editors. Too Close for Comfort: Contentious Issues in Human–Wildlife Encounters. Sydney: Royal Zoological Society of New South Wales; 2008 pp. 73–92.

[10] Robinson AC. The koala in South Australia In: Bergin TJ, editor. The koala: proceedings of the Taronga symposium. Sydney: The Zoological Parks Board of New South Wales; 1978 pp. 132–143.

[11] Phillips B. Koalas: the little Australians we’d all hate to lose. Canberra, Australian Capital Territory; 1990.

[12] Melzer A, Carrick F, Menkhorst P, Lunney D, John BST. Overview, Critical Assessment and Conservation Implications of Koala Distribution and Abundance. 2000;14: 619–628.

[13] Lindsay HA. Re-establishing the koala in South Australia. WildLife. 1950;12: 257–262.

[14] Sherwin WB, Timms P, Wilcken J, Houlden B. Analysis and Conservation Implications of Koala Genetics. Conserv Biol. 2000;14: 639–649. 10.1046/j.1523-1739.2000.99384.x

[15] Lewis F. The koala on Wilsons Promontory. Vic Nat. 1934;51: 76.

[16] Briscoe NJ, Krockenberger A, Handasyde KA, Kearney MR. Bergmann meets Scholander: geographical variation in body size and insulation in the koala is related to climate. J Biogeogr. 2015;42: 791–802. 10.1111/jbi.12445

[17] Martin RW, Handasyde KA, Krockenberger A. Koala In: Van Dyck S, Strahan R, editors. The mammals of Australia. Chatswood, N.S.W.: Australian Museum and Reed Books; 2008 pp. 112–114.

[18] Houlden BA, Costello BH, Sharkey D, Fowler E V, Melzer A, Ellis W, et al Phylogeographic differentiation in the mitochondrial control region in the koala, *Phascolarctos cinereus* (Goldfuss 1817). Mol Ecol. 1999;8: 999–1011. 10.1046/j.1365-294x.1999.00656.x 10434420

[19] McAlpine C, Lunney D, Melzer A, Menkhorst P, Phillips S, Phalen D, et al Conserving koalas: A review of the contrasting regional trends, outlooks and policy challenges. Biol Conserv. 2015;192: 226–236. 10.1016/j.biocon.2015.09.020

[20] Gordon G, Brown AS, Pulsford T. A koala (*Phascolarctos cinereus* Goldfuss) population crash during drought and heatwave conditions in south-western Queensland. Austral Ecol. 1988;13: 451–461. 10.1111/j.1442-9993.1988.tb00993.x

[21] Seabrook L, McAlpine C, Baxter G, Rhodes J, Bradley A, Lunney D. Drought-driven change in wildlife distribution and numbers: a case study of koalas in south west Queensland. Wildl Res. 2011;38: 509 10.1071/WR11064

[22] Adams-Hosking C, McAlpine C, Rhodes JR, Grantham HS, Moss PT. Modelling changes in the distribution of the critical food resources of a specialist folivore in response to climate change. Divers Distrib. 2012;18: 847–860. 10.1111/j.1472-4642.2012.00881.x

[23] McInnes LM, Gillett A, Hanger J, Reid SA, Ryan UM. The potential impact of native Australian trypanosome infections on the health of koalas (*Phascolarctos cinereus*). Parasitology. 2011;138: 873–83. 10.1017/S0031182011000369 21524321

[24] Patterson JLS, Lynch M, Anderson GA, Noormohammadi AH, Legione A, Gilkerson JR, et al The prevalence and clinical significance of Chlamydia infection in island and mainland populations of Victorian koalas (*Phascolartos cinereus*). J Wildl Dis. 2015;51: 309–317. 10.7589/2014-07-176 25588005

[25] Polkinghorne A, Hanger J, Timms P. Recent advances in understanding the biology, epidemiology and control of chlamydial infections in koalas. Vet Microbiol. 2013;165: 214–23. 10.1016/j.vetmic.2013.02.026 23523170

[26] Simmons GS, Young PR, Hanger JJ, Jones K, Clarke D, McKee JJ, et al Prevalence of koala retrovirus in geographically diverse populations in Australia. Aust Vet J. 2012;90: 404–9. 10.1111/j.1751-0813.2012.00964.x 23004234

[27] Dique DS, Thompson J, Preece HJ, Penfold GC, Villiers DL de, Leslie RS. Koala mortality on roads in south-east Queensland: the koala speed-zone trial. Wildl Res. 2003;30: 419 10.1071/WR02029

[28] Lunney D, Gresser S, O’Neill LE, Matthews A, Rhodes J. The impact of fire and dogs on Koalas at Port Stephens, New South Wales, using population viability analysis. Pacific Conserv Biol. 2007;13: 189–201. 10.1071/PC070189

[29] Department of the Environment. *Phascolartos cinereus* (combined populations of Queensland, New South Wales and the Australian Capital Territory) in species profile and threats database Canberra, Australian Capital Territory: Department of the Environment; 2016 Available: http://www.environment.gov.au/cgi-bin/sprat/public/publicspecies.pl?taxon_id=85104

[30] Masters P, Duka T, Berris S, Moss G. Koalas on Kangaroo Island: from introduction to pest status in less than a century. Wildl Res. 2004;31: 267 10.1071/WR03007

[31] Middleton DR, Walters B, Menkhorst P, Wright P. Fertility control in the koala, *Phascolarctos cinereus*: the impact of slow-release implants containing levonorgestrel or oestradiol on the production of pouch young. Wildl Res. 2003;30: 207 10.1071/WR02052

[32] Hynes EF, Handasyde KA, Shaw G, Renfree MB. Levonorgestrel, not etonogestrel, provides contraception in free-ranging koalas. Reprod Fertil Dev. 2010;22: 913–9. 10.1071/RD09253 20591325

[33] Whisson DA, Dixon V, Taylor ML, Melzer A. Failure to Respond to Food Resource Decline Has Catastrophic Consequences for Koalas in a High-Density Population in Southern Australia. PLoS One. 2016;11: e0144348 10.1371/journal.pone.0144348 26735846 PMC4703219

[34] Lee KE, Seddon JM, Johnston S, FitzGibbon SI, Carrick F, Melzer A, et al Genetic diversity in natural and introduced island populations of koalas in Queensland. Aust J Zool. 2012;60: 303 10.1071/ZO12075

[35] Ruiz-Rodriguez CT, Ishida Y, Greenwood AD, Roca AL. Development of 14 microsatellite markers in the Queensland koala (*Phascolarctos cinereus adustus*) using next generation sequencing technology. Conserv Genet Resour. 2014;6: 429–431. 10.1007/s12686-013-0115-2 25067980 PMC4109682

[36] Taylor AC, Graves JAM, Murray ND, Sherwin WB. Conservation genetics of the koala (*Phascolarctos cinereus*) II. Limited variability in minisatellite DNA sequences. Biochem Genet. 1991;29: 355–363. 10.1007/BF00554143 1747097

[37] Kjeldsen SR, Zenger KR, Leigh K, Ellis W, Tobey J, Phalen D, et al Genome-wide SNP loci reveal novel insights into koala (*Phascolarctos cinereus*) population variability across its range. Conserv Genet. 2015;17: 337–353. 10.1007/s10592-015-0784-3

[38] Tsangaras K, Avila-Arcos MC, Ishida Y, Helgen KM, Roca AL, Greenwood AD, et al Historically low mitochondrial DNA diversity in koalas (*Phascolarctos cinereus*). BMC Genet. 2012;13: 92 10.1186/1471-2156-13-92 23095716 PMC3518249

[39] Houlden BA, England PR, Taylor AC, Greville WD, Sherwin WB. Low genetic variability of the koala Phascolarctos cinereus in south-eastern Australia following a severe population bottleneck. Mol Ecol. 1996;5: 269–281. 10.1046/j.1365-294X.1996.00089.x 8673272

[40] Seymour AM, Montgomery ME, Costello BH, Ihle S, Johnsson G, St John B, et al High effective inbreeding coefficients correlate with morphological abnormalities in populations of South Australian koalas (*Phascolarctos cinereus*). Anim Conserv. 2001;4: 211–219. 10.1017/S1367943001001251

[41] Fowler E V., Houlden BA, Hoeben P, Timms P. Genetic diversity and gene flow among southeastern Queensland koalas (*Phascolarctos cinereus*). Mol Ecol. 2000;9: 155–164. 10.1046/j.1365-294x.2000.00844.x 10672159

[42] Taylor AC, Graves JAM, Murray ND, O’Brien SJ, Yuhki N, Sherwin WB. Conservation genetics of the koala (*Phascolarctos cinereus*): low mitochondrial DNA variation amongst southern Australian populations. Genet Res. 1997;69: 25–33. Available: http://journals.cambridge.org/abstract_S0016672397002607 9164173 10.1017/s0016672397002607

[43] Cristescu R, Cahill V, Sherwin WB, Handasyde K, Carlyon K, Whisson D, et al Inbreeding and testicular abnormalities in a bottlenecked population of koalas (*Phascolarctos cinereus*). Wildl Res. 2009;36: 299 10.1071/WR08010

[44] Lee T, Zenger KR, Close RL, Jones M, Phalen DN, A TL, et al Defining spatial genetic structure and management units for vulnerable koala (*Phascolarctos cinereus*) populations in the Sydney region, Australia. Wildl Res. 2010;37: 156 10.1071/WR09134

[45] Lee KE, Seddon JM, Corley SW, Ellis WAH, Johnston SD, de Villiers DL, et al Genetic variation and structuring in the threatened koala populations of Southeast Queensland. Conserv Genet. 2010;11: 2091–2103. 10.1007/s10592-009-9987-9

[46] Lee T, Zenger KR, Close RL, Phalen DN. Genetic analysis reveals a distinct and highly diverse koala (*Phascolarctos cinereus*) population in South Gippsland, Victoria, Australia. Aust Mammal. 2012;34: 68 10.1071/AM10035

[47] Cristescu R, Sherwin WB, Handasyde K, Cahill V, Cooper DW. Detecting bottlenecks using BOTTLENECK 1. 2. 02 in wild populations: the importance of the microsatellite structure. 2010; 1043–1049. 10.1007/s10592-009-9949-2

[48] Houlden BA, England P, Sherwin WB. Paternity Exclusion in Koalas Using Hypervariable Microsatellites. J Hered. 1996;87: 149–152. 10.1093/oxfordjournals.jhered.a022972 8830092

[49] Dennison S, Frankham GJ, Neaves LE, Flannagan C, Fiztgibbon S, Eldridge MDB, et al Population genetics of the koala (*Phascolarctos cinereus*) in Northeastern New South Wales and Southeastern Queensland. Aust J Zool. Submitted;

[50] Sunnucks P, Hale P. Numerous transposed sequences of mitochondrial cytochrome oxidase I-II in aphids of the genus *Sitobion* (Hemiptera: Aphididae). Mol Biol Evol. 1996;13: 510–524. 8742640 10.1093/oxfordjournals.molbev.a025612

[51] Fumagalli L, Pope LC, Taberlet P, Moritz C. Versatile primers for the amplification of the mitochondrial DNA control region in marsupials. Mol Ecol. 1997;6: 1199–1201. 9421920 10.1046/j.1365-294x.1997.00298.x

[52] Tsangaras K, Wales N, Sicheritz-Pontén T, Rasmussen S, Michaux J, Ishida Y, et al Hybridization capture using short PCR products enriches small genomes by capturing flanking sequences (CapFlank). PLoS One. 2014;9: e109101 10.1371/journal.pone.0109101 25275614 PMC4183570

[53] Kearse M, Moir R, Wilson A, Stones-Havas S, Cheung M, Sturrock S, et al Geneious Basic: an integrated and extendable desktop software platform for the organization and analysis of sequence data. Bioinformatics. 2012;28: 1647–9. 10.1093/bioinformatics/bts199 22543367 PMC3371832

[54] Tamura K, Stecher G, Peterson D, Filipski A, Kumar S. MEGA6: Molecular Evolutionary Genetics Analysis Version 6.0. Mol Biol Evol. 2013;30: 2725–2729. 10.1093/molbev/mst197 24132122 PMC3840312

[55] Guindon S, Gascuel O. A Simple, Fast, and Accurate Algorithm to Estimate Large Phylogenies by Maximum Likelihood. Syst Biol. 2003;52: 696–704. 10.1080/10635150390235520 14530136

[56] Posada D. jModelTest: phylogenetic model averaging. Mol Biol Evol. 2008;25: 1253–6. 10.1093/molbev/msn083 18397919

[57] Seddon JM, Lee KE, Johnston SD, Nicolson VN, Pyne M, Carrick FN, et al Testing the regional genetic representativeness of captive koala populations in South-East Queensland. Wildl Res. 2014;41: 277 10.1071/WR13103

[58] Bazinet AL, Zwickl DJ, Cummings MP. A gateway for phylogenetic analysis powered by grid computing featuring GARLI 2.0. Syst Biol. 2014;63: 812–8. 10.1093/sysbio/syu031 24789072 PMC4141202

[59] Drummond AJ, Suchard MA, Xie D, Rambaut A. Bayesian phylogenetics with BEAUti and the BEAST 1.7. Mol Biol Evol. 2012;29: 1969–73. 10.1093/molbev/mss075 22367748 PMC3408070

[60] Drummond AJ, Rambaut A. BEAST: Bayesian evolutionary analysis by sampling trees. BMC Evol Biol. BioMed Central; 2007;7: 214 10.1186/1471-2148-7-214 17996036 PMC2247476

[61] Hasegawa M, Kishino H, Yano T. Dating of the human-ape splitting by a molecular clock of mitochondrial DNA. J Mol Evol. 1985;22: 160–174. 10.1007/BF02101694 3934395

[62] Van Dyck S, Strahan R. The Mammals of Australia., 3rd edn. Reed New Holland: Sydney 2008.

[63] Clement M, Snell Q, Walker P, Posada D, Crandall K. TCS: Estimating Gene Genealogies. Parallel and Distributed Processing Symposium, International Proceedings. 2002. p. 184. Available: http://www.computer.org/csdl/proceedings/ipdps/2002/1573/02/15730184.pdf

[64] Templeton AR, Crandall KA, Sing CF. A cladistic analysis of phenotypic associations with haplotypes inferred from restriction endonuclease mapping and DNA sequence data. III. Cladogram estimation. Genetics. 1992;132: 619–633. Available: http://www.genetics.org/content/132/2/619.full.pdf+html 1385266 10.1093/genetics/132.2.619 PMC1205162

[65] Excoffier L, Lischer HEL. Arlequin suite ver 3.5: a new series of programs to perform population genetics analyses under Linux and Windows. Mol Ecol Resour. 2010;10: 564–7. 10.1111/j.1755-0998.2010.02847.x 21565059

[66] Fu YX. Statistical Tests of Neutrality of Mutations Against Population Growth, Hitchhiking and Background Selection. Genetics. 1997;147: 915–925. Available: http://www.genetics.org/content/147/2/915.short 9335623 10.1093/genetics/147.2.915 PMC1208208

[67] Tajima F. Statistical method for testing the neutral mutation hypothesis by DNA polymorphism. Genetics. 1989;123: 585–595. Available: http://www.genetics.org/content/123/3/585.short 2513255 10.1093/genetics/123.3.585 PMC1203831

[68] Dupanloup I, Schneider S, Excoffier L. A simulated annealing approach to define the genetic structure of populations. Mol Ecol. 2002;11: 2571–2581. 10.1046/j.1365-294X.2002.01650.x 12453240

[69] Jensen JL, Bohonak AJ, Kelley ST. Isolation by distance, web service. BMC Genet. 2005;6: 13 10.1186/1471-2156-6-13 15760479 PMC1079815

[70] Rousset F. Genetic Differentiation and Estimation of Gene Flow from F-Statistics Under Isolation by Distance. Genetics. 1997;145: 1219–1228. Available: http://www.genetics.org/content/145/4/1219.short 9093870 10.1093/genetics/145.4.1219 PMC1207888

[71] Schneider S, Excoffier L. Estimation of Past Demographic Parameters From the Distribution of Pairwise Differences When the Mutation Rates Vary Among Sites: Application to Human Mitochondrial DNA. Genetics. 1999;152: 1079–1089. 10388826 10.1093/genetics/152.3.1079 PMC1460660

[72] Woinarski JCZ, Burbidge AA, Harrison PL. Action Plan for Australian Mammals 2012. Collingwood, Victoria: CSIRO Publishing; 2014 Available: http://www.publish.csiro.au/pid/7010.htm

[73] Ramírez-Soriano A, Ramos-Onsins SE, Rozas J, Calafell F, Navarro A. Statistical power analysis of neutrality tests under demographic expansions, contractions and bottlenecks with recombination. Genetics. Genetics; 2008;179: 555–67. 10.1534/genetics.107.083006 18493071 PMC2390632

[74] Moritz C. Defining “Evolutionarily Significant Units” for conservation. Trends Ecol Evol. 1994;9: 373–5. 10.1016/0169-5347(94)90057-4 21236896

[75] Bryant LM, Fuller SJ. Pleistocene climate fluctuations influence phylogeographical patterns in *Melomys cervinipes* across the mesic forests of eastern Australia. J Biogeogr. 2014;41: 1923–1935. 10.1111/jbi.12341

[76] McGuigan K, McDonald K, Parris K, Moritz C. Mitochondrial DNA diversity and historical biogeography of a wet forest-restricted frog (*Litoria pearsoniana*) from mid-east Australia. Mol Ecol. 1998;7: 175–186. 10.1046/j.1365-294x.1998.00329.x 9532760

[77] Keogh SJ, Scott IAW, Fitzgerald M, Shine R. Phylogeographic patterns in reptiles on the New England Tablelands at the south-western boundaryof the McPherson Macleay Overlap. Conserv Genet. 2003;4: 57–65. 10.1023/A:1021823423944

[78] Rix MG, Harvey MS. Phylogeny and historical biogeography of ancient assassin spiders (Araneae: Archaeidae) in the Australian mesic zone: evidence for Miocene speciation within Tertiary refugia. Mol Phylogenet Evol. 2012;62: 375–96. 10.1016/j.ympev.2011.10.009 22040763

[79] Hazlitt SL, Goldizen AW, Nicholls JA, Eldridge MDB. Three divergent lineages within an Australian marsupial (*Petrogale penicillata*) suggest multiple major refugia for mesic taxa in southeast Australia. Ecol Evol. 2014;4: 1102–16. 10.1002/ece3.1009 24772286 PMC3997325

[80] Rowe KMC, Rowe KC, Elphinstone MS, Baverstock PR. Population structure, timing of divergence and contact between lineages in the endangered Hastings River mouse (*Pseudomys oralis*). Aust J Zool. 2011;59: 186 10.1071/ZO11046

[81] Colgan DJ, O’Meally D, Sadlier RA. Phylogeographic patterns in reptiles on the New England Tablelands at the south-western boundary of the McPherson Macleay Overlap. Aust J Zool. 2009;57: 317 10.1071/ZO08088

[82] Edwards DL, Melville J. Phylogeographic analysis detects congruent biogeographic patterns between a woodland agamid and Australian wet tropics taxa despite disparate evolutionary trajectories. J Biogeogr. 2010; 10.1111/j.1365-2699.2010.02293.x

[83] Chapple DG, Hoskin CJ, Chapple SNJ, Thompson MB. Phylogeographic divergence in the widespread delicate skink (*Lampropholis delicata*) corresponds to dry habitat barriers in eastern Australia. BMC Evol Biol. 2011;11: 191 10.1186/1471-2148-11-191 21726459 PMC3141439

[84] Dique DS, Thompson J, Preece HJ, Villiers DL de, Carrick FN. Dispersal patterns in a regional koala population in south-east Queensland. Wildl Res. 2003;30: 281 10.1071/WR02043

[85] Ellis WAH, Melzer A, Carrick FN, Hasegawa M. Tree use, diet and home range of the koala (*Phascolarctos cinereus*) at Blair Athol, central Queensland. Wildl Res. 2002;29: 303 10.1071/WR00111

[86] Adams-Hosking C, Moss P, Rhodes J, Grantham H, McAlpine C. Modelling the potential range of the koala at the Last Glacial Maximum: future conservation implications. Aust Zool. 2011;35: 983–990. 10.7882/AZ.2011.052

[87] Grant WS, Liu M, Gao T, Yanagimoto T. Limits of Bayesian skyline plot analysis of mtDNA sequences to infer historical demographies in Pacific herring (and other species). Mol Phylogenet Evol. 2012;65: 203–12. 10.1016/j.ympev.2012.06.006 22750109

[88] de Groot GA, Nowak C, Skrbinšek T, Andersen LW, Aspi J, Fumagalli L, et al Decades of population genetic research reveal the need for harmonization of molecular markers: the grey wolf *Canis lupus* as a case study. Mamm Rev. 2016;46: 44–59. 10.1111/mam.12052

[89] Pereira L, Van Asch B, Amorim A. Standardisation of nomenclature for dog mtDNA D-loop: a prerequisite for launching a *Canis familiaris* database. Forensic Sci Int. 2004;141: 99–108. 10.1016/j.forsciint.2003.12.014 15062947

[90] Andrews RM, Kubacka I, Chinnery PF, Lightowlers RN, Turnbull DM, Howell N. Reanalysis and revision of the Cambridge reference sequence for human mitochondrial DNA. Nat Genet. 1999;23: 147 10.1038/13779 10508508

